# Rereplication in *emi1*-Deficient Zebrafish Embryos Occurs through a Cdh1-Mediated Pathway

**DOI:** 10.1371/journal.pone.0047658

**Published:** 2012-10-17

**Authors:** Mara E. Robu, Yong Zhang, Jennifer Rhodes

**Affiliations:** Immune Cell Development and Host Defense Program, Fox Chase Cancer Center, Temple University Health System, Philadelphia, Pennsylvania, United States of America; University of Minnesota, United States of America

## Abstract

Disruption of early mitotic inhibitor 1 (Emi1) interferes with normal cell cycle progression and results in early embryonic lethality in vertebrates. During S and G2 phases the ubiquitin ligase complex APC/C is inhibited by Emi1 protein, thereby enabling the accumulation of Cyclins A and B so they can regulate replication and promote the transition from G2 phase to mitosis, respectively. Depletion of Emi1 prevents mitotic entry and causes rereplication and an increase in cell size. In this study, we show that the developmental and cell cycle defects caused by inactivation of zebrafish *emi1* are due to inappropriate activation of APC/C through its cofactor Cdh1. Inhibiting/slowing progression into S-phase by depleting Cdt1, an essential replication licensing factor, partially rescued *emi1* deficiency-induced rereplication and the increased cell size. The cell size effect was enhanced by co-depletion of cell survival regulator *p53*. These data suggest that the increased size of *emi1*-deficient cells is either directly or indirectly caused by the rereplication defects. Moreover, enforced expression of Cyclin A partially ablated the rereplicating population in *emi1*-deficient zebrafish embryos, consistent with the role of Cyclin A in origin licensing. Forced expression of Cyclin B partially restored the G1 population, in agreement with the established role of Cyclin B in mitotic progression and exit. However, expression of Cyclin B also partially inhibited rereplication in *emi1-*deficient embryos, suggesting a role for Cyclin B in regulating replication in this cellular context. As Cyclin A and B are substrates for APC/C-Cdh1 - mediated degradation, and Cdt1 is under control of Cyclin A, these data indicate that *emi1* deficiency-induced defects *in vivo* are due to the dysregulation of an APC/C-Cdh1 molecular axis.

## Introduction

Early mitotic inhibitor 1 (Emi1) is a cell cycle regulator that is essential for proper progression through cell cycle [1]. EMI1 is regulated in a cell cycle-dependent manner, wherein *EMI1* gene transcription is activated upon entry into S-phase by E2F2 and the protein is phosphorylated and degraded early in mitosis [1], [2], [3], [4], [5]. As such, EMI1 is present in Ki-67-positive proliferating cells in a variety of adult murine tissues [6], [7]. Studies of the mammalian and *Xenopus* homologues of EMI1 have shown that it inhibits the Anaphase-Promoting Complex/Cyclosome (APC/C), an ubiquitin ligase complex that targets cell cycle regulated proteins such as the S- and G2-phase Cyclins A and B, Securin and Geminin [1], [2], [8], [9]. Thus, the release of APC/C from EMI1 inhibition during mitosis allows for the ubiquitination and degradation of these key substrates and promotes progression through mitosis [5], [10].

EMI1 is essential to regulate progression through the cell cycle. Depletion of *EMI1* by siRNA knockdown in human cell lines or immunodepletion in cycling *Xenopus* extracts results in the untimely degradation of APC/C substrates, leading to a G2/M arrest and inducing rereplication [1], [8], [11], [12]. Analysis by microscopy showed that knockdown of *EMI1* in human cell lines prevented chromosome condensation and nuclear membrane breakdown, indicating that *EMI1*-deficient cells are blocked in G2 and do not proceed into mitosis [1], [11].

*EMI1*-deficient human cell lines and *emi1*-deficient zebrafish embryos display an increase in nuclear and whole cell size [11], [12], [13]. Flow cytometry analysis revealed that the *EMI1* depletion-induced increase in ploidy in cells actively replicating their DNA correlates with enlarged nuclei [11]. However, it is not clear whether the increase in cell and nuclear size is a consequence of rereplication, prolonged cell cycle arrest or the misregulation of a growth pathway in which the activity of Emi1 has not been previously linked.

APC/C binds to the cofactor Cdc20 early in mitosis and transitions to using the Cdh1 cofactor in late mitosis and through G1 phase [14]; however, both Cdh1 and Cdc20 promote the degradation of Cyclins A and B [15], [16]. Rereplication in *EMI1*-depleted human cell lines was partially inhibited by co-depletion of APC/C cofactors *CDH1*, *CDC20* or the addition of a non-degradable form of Cyclin A [11], [12]. Similarly, Di Fiore and Pines examined the progression through a single cell division in synchronized HeLa cells to show that cells depleted of both *EMI1* and *CDH1* progressed through S and G2/M stages with similar kinetics to control cells, while *EMI1*-deficient cells were delayed in G2/M [11]. Interestingly, the cell cycle distribution in synchronously dividing *EMI1*-deficient HeLa cells was only restored back to a wild-type distribution upon depletion of both *CDC20* and *CDH1* [12], suggesting that in some contexts the activity of CDC20 may contribute to *EMI1* depletion-mediated defects. It remains to be examined in a more complex biological system whether Cdh1 and Cyclin A are the key components regulating events downstream of Emi1 depletion, or if Cdc20 and Cyclin B are also important contributors. However, these studies are complicated by the essential nature of cell cycle regulation during embryonic development.

Mutation in the *Drosophila EMI1* homologue *rca1* prevents mitotic entry during early embryonic development and in the imaginal disk [17]. In vertebrates, *Emi1* mutation results in very early embryonic lethality in mice due to severe mitotic defects and increased apoptosis prior to zygote implantation [7]. Recent studies using the zebrafish model system showed that mutation or antisense morpholino-mediated knockdown of *emi1* leads to defects in morphogenesis and an inhibition of cell division [13], [18], [19]. However, *emi1*-deficient zebrafish embryos survive beyond the stage when body patterning and many organ systems are established [13], [18], [19], which is likely due to the rapid development of the zebrafish larvae and maternal expression of *emi1* [13]. Embryos homozygous for truncated mutant forms of *emi1* (ti121, ti245, x1) display a loss of phosphorylated-Histone H3 (pH3)-positive mitotic cells during early gastrulation and have robust morphological defects [18], [19], whereas mutants harboring a hypomorphic allele (hi2618) displayed less severe developmental defects, retained pH3 positive cells through somitogenic stages, but showed decreased numbers of hematopoietic cells and total DAPI-stained nuclei in the trunk region [13]. Interestingly, both severe and hypomorphic mutations of *emi1* lead to embryos with increased BrdU incorporation at 24 hours post-fertilization (hpf), suggesting that even a partial loss of *emi1* causes defects in the regulation of replication [13], [18], [19]. Rereplication is most likely the cause of increased ploidy observed in zebrafish *emi1* mutant metaphases [13], consistent with the flow cytometric detection of increased DNA content seen in zebrafish cells and human cell lines depleted of *EMI1* [11], [12], [13].

DNA content has been evaluated as a potential factor contributing to cell size [20]. Zebrafish embryos deficient in *emi1* showed increased cell size by flow cytometry [13] and increased nuclear size by DAPI staining [13], [18], consistent with previous cell line data [11], [12]. The morphology and cell cycle defects of *emi1* ^hi2648^ mutants were not altered by the absence of p53 activity [13]. In total, these data establish that zebrafish is an effective model to examine the *in vivo* relationship between *emi1* and downstream effectors.

In this study, we dissected the genetic pathway through which loss of *emi1* exerts it effects in developing zebrafish embryos. First, we determined the developmental age at which newly spliced (predominantly zygotic) *emi1* transcripts are required to prevent cell cycle defects. We then dissected the phenotype of *emi1* deficiency by manipulating the levels of factors hypothesized to have aberrant activity in this cellular context. This analysis makes three important findings. First, embryos deficient for *emi1* can be restored back to a wild-type phenotype by antisense morpholino inhibition of the APC/C cofactor *cdh1*, suggesting that *in vivo*, Cdh1-mediated degradation of substrates is responsible for the *emi*-deficient phenotype. Second, partially inhibiting origin licensing by *cdt1* knockdown [21] ablated the rereplicating phenotype of *emi1*-deficient cells and normalized the cell size in these embryos, thereby linking these phenotypes. Lastly, enforced expression of either Cyclin A or Cyclin B, could partially rescue the rereplication defects in *emi1*-deficient embryos, supporting a less well-known role of Cyclin B in regulating replication *in vivo*. Given the role of Cdh1 in targeting Cyclins A and B for degradation [16], this study provides strong evidence that a Cdh1 axis is responsible for the rereplication and increased cell size in *emi1*-deficient embryos.

## Results

### Emi1-deficiency-induced Defects are Due to APC/C-Cdh1 Activity

**Figure 1:**
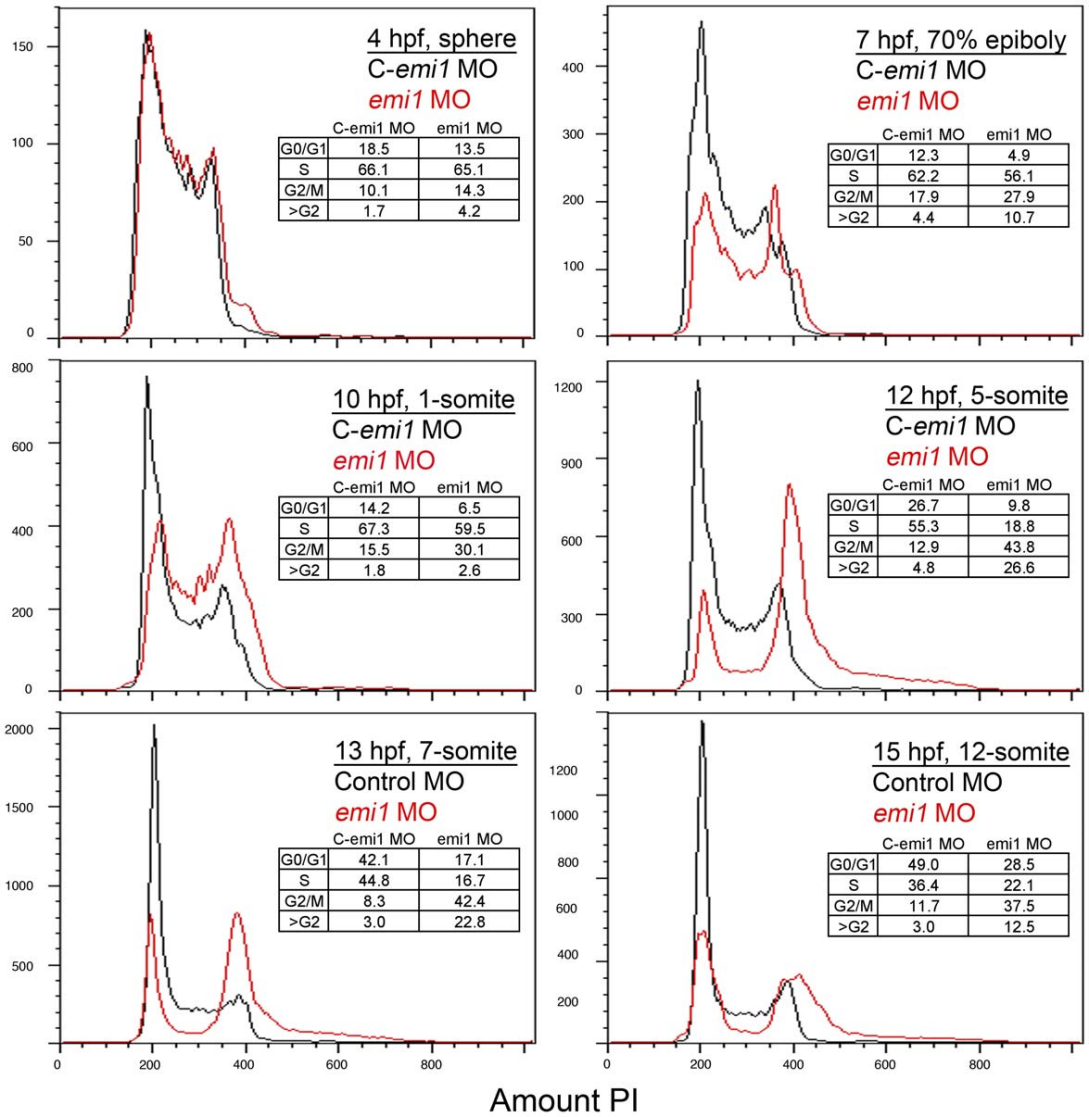
Developmental time course of *emi1* morphant cell cycle defects. FACS scanning of propidium iodide (PI)-stained total cells from embryos injected with control (C, in black) or *emi1*-specific morpholinos (in red) (2 ng per embryo). Each panel shows an overlay of the distribution of control and *emi1*-morphant cells. Age and developmental stage of embryos is indicated. The insert shows the percent of cells with 2 n DNA content (G0/G1), cells replicating their DNA (S), cells with 4 n DNA content (G2/M) and cells with greater than 4 n DNA content. The percent of cells in each stage has been estimated with the Watson mathematical model in Flowjo software, except for the 4 hpf and 7 hpf time points for each we have assigned the gates for each cell cycle stage.

To gain insights into the developmental stage at which depletion of *emi1* affects the cell cycle, we used morpholino oligonucleotide to block *emi1* splicing. Following fertilization of the egg, zebrafish zygotic cells rapidly divide and do not have gap phases in the cell cycle. Asynchronous cell proliferation begins during the mid-blastula transition around 3 hpf. During this period, the cell cycle lengthens and transcription is activated [22]. Emi1 is maternally expressed and, thus, we used the morpholino to determine the age at which the embryo becomes dependent on newly spliced *emi1* transcripts and to define the earliest *emi1* depletion-induced defects in the cell cycle. The *emi1* morpholino obstructs splicing as expected, although it does not completely deplete wild-type *emi1* transcripts (Fig. S1 A). Propidium iodide-based analysis of the cell cycle over a developmental time course showed indistinguishable distributions in 4 hpf cells from control-injected or *emi1* morpholino-injected (morphant) embryos (Fig. 1). However, by 7 hpf, the *emi1*-depleted population showed slightly decreased number of cells with 2 n content of DNA (G0/G1 cells) and an increased numbers of cells with 4 n amount of DNA (G2/M cells), in comparison to the control cell population. Between 10 and 12 hpf, during early somitogenesis, the defects in the *emi1* morphant cell cycle become more severe, with a robust increase in 4 n cells and the accumulation of cells with greater than 4 n DNA content indicative of rereplication. This result is consistent with our previous work showing that the hypomorphic *emi1* allele hi2648 causes rereplication [13]. The number of cells with 4 n and more than 4 n DNA content in *emi1* morphant embryos decreases over time, likely due to the increase in cell death of cells undergoing rereplication [23]. Therefore, we chose to perform the next cell cycle experiments at 10 to 12 hpf (4–5 somites) when we see the peak of cell cycle defects in *emi1* morphant cells.

**Figure 2:**
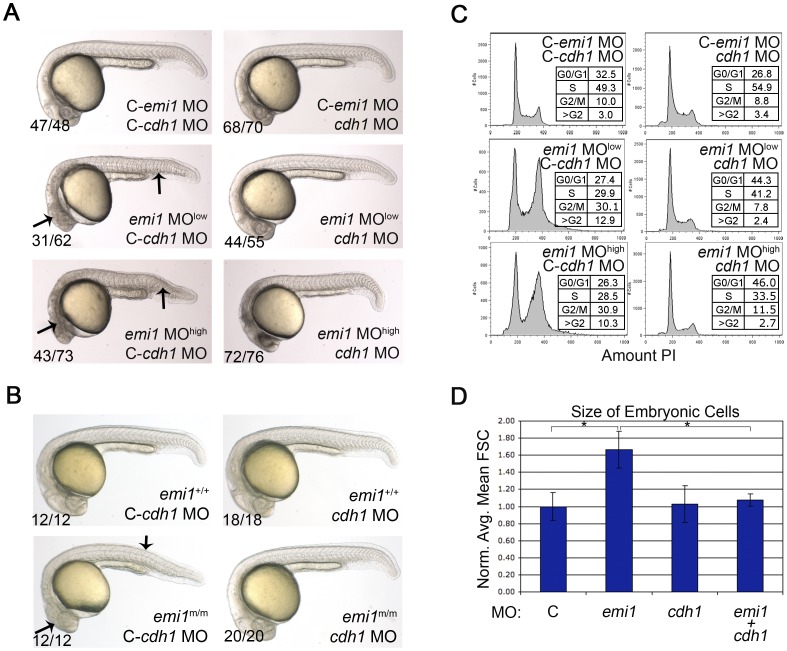
Cellular and developmental defects caused by *emi1* knockdown are due to *cdh1* activity. (A and B) Phase-contrast analysis of embryos at 24 hpf. (A) Morpholino injected embryos, where C- indicates the mismatch control morpholino for the indicated gene. We injected 1 ng of control and *cdh1* morpholinos per embryo. The *emi1* morpholino was injected at 1 ng (low) or 2.7 ng (high) per embryo. (B) Genotyped wild-type siblings (+/+) and homozygous *emi1* mutant embryos (m/m) injected with 2 ng control or *cdh1* morpholinos as indicated. Note the wild-type appearance of homozygous mutants and *emi1* morphants that were injected with *cdh1* morpholino. (C) Cell cycle analysis by FACS scanning of propidium iodide (PI)-stained cells. Total cells were analyzed from single cell suspensions of pools of the indicated morpholino-injected embryos at 4–5 somites. A representative experiment is shown. (D) The average cell size from FACS analysis of cells from morpholino-injected disaggregated embryos at 4–5 somites, normalized to the average control cell size (C, arbitrarily set at 1.00). Error bars indicate standard deviation (SD). Cell size data and SD were obtained from 3 independent experiments.

We next examined whether the *emi1* deficiency-induced defects are due to the activity of APC/C. To inhibit APC/C we used morpholinos to knockdown the APC/C cofactors *cdc20* and *cdh1*. Knockdown of *cdc20* was severely lethal to the zebrafish embryos, alone or in combination with *emi1* knockdown (data not shown), consistent with *cdc20* being an essential gene in yeast [24], [25] and mice [26]. On the other hand, *cdh1* is not essential for embryogenesis [27] and knockdown of this gene in zebrafish did not result in any overt morphological or cell cycle defects (Fig. 2). Therefore we focused our analysis on the effects of *cdh1* on *emi1*-deficient embryos. As previously described, at 24 hpf the morphological defects caused by *emi1* depletion include small heads, with cell death in the head, abnormal somite structure and ventral tail curvature. Using two different doses of *emi1* morpholino, we found that co-knockdown of *emi1* and *cdh1* gave rise to fully or partially rescued embryos based on visual assessment of embryonic morphology (Fig. 2 A). Consistent with this finding, we injected control or *cdh1* morpholinos into a clutch of embryos generated from breeding *emi1* heterozygous parents and then photographed and genotyped each individual embryo. All 20 of the homozygous mutant embryos (out of a clutch of 77 embryos) exhibited a wild-type appearance when injected with *cdh1* morpholino. Representative *emi1* mutant embryos and siblings are shown in Fig. 2 B. The striking reversion of the *emi1* phenotype suggests that much, if not all, of the developmental defects are a result of APC/C-Cdh1 activity.

With this in mind, we examined whether the cell cycle defects in *emi1* morphant cells are due to *cdh1* function. Indeed, at 12 hpf, cells co-depleted for *cdh1* and *emi1* showed a normal cell cycle distribution (Fig. 2 C). Rereplication induces increased levels of phosphorylated Histone H2AX (pH2AX), a marker for DNA damage [28], [29]. Depletion of *emi1* causes increased levels of pH2AX as detected by Western and this level is brought back to normal by co-depletion of *cdh1* (Fig. S1 B). Our lab and others have previously correlated the *emi1* depletion-induced cell cycle defects with an increase in cell size [11], [12], [13], [18]. Building on this information, we examined the average size of cycling cells from 3 independent experiments and found that *emi1* depletion caused more than a 1.5 fold increase in size, consistent with previous reports. In this context, *cdh1* knockdown rescued the cell size back to normal (Fig. 2 D). A second independent *cdh1* morpholino showed similar effects on morphology and cell cycle defects in *emi1* morphants (data not shown). In total, these data indicate that the developmental and cell cycle defects due to the loss of *emi1* are caused by the activation of APC/C-Cdh1.

### Rereplication Underlies Large Cell Phenotype in *emi1*-deficient Embryos

**Figure 3:**
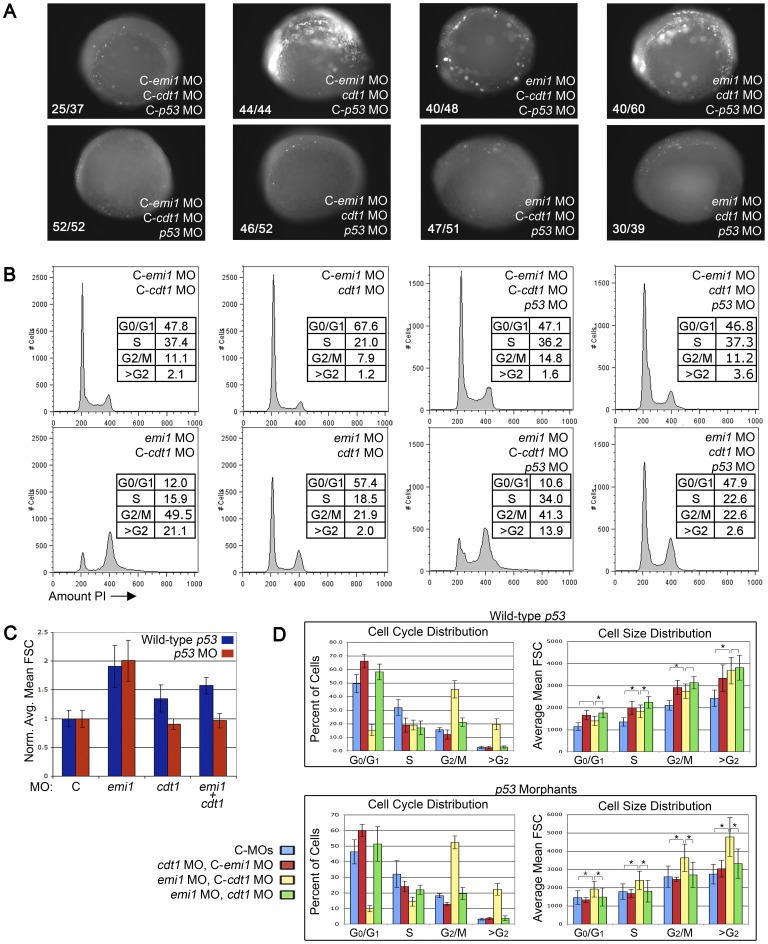
Knockdown of *cdt1* partially ablates rereplication and increased cell size in *emi1* morphants. (A) Cell death assay in 4–5 somite embryos injected with the indicated morpholinos. C- indicates the mismatch control morpholino for the indicated gene. We injected 2 ng *emi1* MO or C-*emi1* MO, 2.7 ng *cdt1* MO or C-*cdt1* MO, 7 ng *p53* MO or C-*p53* MO per embryo from a cocktail mix. Cell death was detected by immunoflurescence staining of activated Caspase 3. Note the high levels of activated Caspase 3 in *cdt1* morphants and *cdt1 emi1* double morphants. Co-knockdown of *p53* significantly alleviated the cell death in all cases. (B) Representative PI-based cell cycle analysis of total cells from pools of 4–5 somite embryos injected with the indicated morpholinos (same doses as in A). Note that *cdt1* morpholino partially, but significantly rescued the cell cycle defects in *emi1* morphants. The 4 panels on the right show no significant effect of *p53* knockdown on cell cycle distribution in embryos injected with *cdt1* or *emi1* morpholinos or both. (C) Normalized average cell size based on FACS analysis of total cells from the indicated morphants from 10 independent experiments. We removed the highest and lowest value for each sample and averaged data from 8 experiments. (D) Summary of the cell cycle and cell size distribution at different phases of the cell cycle. Top panels were obtained from *p53* wild-type embryos, bottom panels show data from *p53* morphant embryos. The legend indicates the morphant populations.

Having demonstrated that *cdh1* loss reverts the *emi1*-deficiency-induced defects, we sought to further examine the pathways that could contribute to the Cdh1-mediated events. The activity of APC/C in *emi1*-deficient human cells leads to the untimely degradation of APC/C targets Cyclins A and B as well as Geminin, an S-phase inhibitor of the pre-replicative complex [1], [8], [11], [12]. The pre-replicative complex is formed in early G1 as the replication origins are bound by licensing factors Cdc6 and Cdt1, which recruit helicase proteins (MCMs) to unwind of the DNA – reviewed in [30]. To prevent the replication origins from firing more than once in a single S phase, when replication begins, the Cdc6 and Cdt1 proteins are phosphorylated by Cyclin A-dependent kinases, and then Cdt1 is targeted for degradation and Cdc6 is exported to the cytoplasm - reviewed in [31]. Thus, it is likely that Cdc6 and Cdt1 remain active in *emi1*-deficient cells due to low Cyclin A levels and they facilitate the erroneous re-firing of the origins of replication. A morpholino targeting *cdt1* has been described [32] and inhibits normal *cdt1* splicing (Fig. S2 C). Due to the essential role of Cdt1 in the pre-replicative complex, we expected *cdt1* knockdown to result in severe developmental defects, significant cell death (Fig. 3 A) and an increased percent of cells in G1 phase with a corresponding decrease in the numbers of cells in S and G2/M phases (Fig. 3 B). Indeed, by 24 hpf, *cdt1* morphants showed severely increased, and widespread, cell death and had small heads and a shortened anterior-posterior body axis compared to control-injected embryos (Fig. S2 A). Interestingly, the developmental defects appeared more severe in *emi1*/*cdt1* co-depleted embryos compared to embryos depleted of either single gene, which may be due to their unique roles regulating different aspects of cell cycle progression and/or the combinatorial induction of cell death.

Developmental defects due to a blockade in cell cycle progression and increased cell death effects of the *cdt1* morpholino could result from *p53* activation [33]. Previously, we have shown that the cell cycle defects in *emi1*-deficient zebrafish cells are *p53*-independent, while the increased embryonic cell death was *p53*-dependent [13]. Here, RT-PCR analysis showed that there were no differences in the knockdown of *cdt1* and/or *emi1* transcript levels in the presence or absence of a *p53* morpholino (Fig. S2 C). We found that embryos co-depleted for *cdt1* and *p53* showed significantly less cell death as assayed by activated Caspase 3 staining at 5-somites (Fig. 3 A) and less severe morphological defects than *cdt1* morphants with wild-type *p53* activity at 24hpf (Fig. S2 A). However, the *cdt1*/*p53* and *cdt1*/*p53*/*emi1* depleted embryos continued to display developmental defects, indicating that some but not all of the *cdt1* morpholino-induced defects are mediated by *p53*. Of note, while *p53* knockdown ablated the cell death in *cdt1* morphants at 5-somites, by 24 hpf the co-depleted embryos displayed decreased but evident cell death compared to *cdt1*-morphants. This is consistent with Cdt1 being essential for cell survival and suggests that the cell death caused by *cdt1* knockdown is initially p53-dependent, but later on p53-independent cell death mechanisms may start to come into play, due to sustained inability of cells to initiate replication.

Not surprisingly, we found that embryos co-injected with morpholinos inhibiting *cdt1*, *emi1* and *p53* appeared to have less severe developmental defects with decreased amount of dead tissue compared to embryos with normal *p53* function. The *cdt1*/*p53*/*emi1* morphants displayed robust developmental defects which appeared less severe than *cdt1*/*p53* morphants and more severe than *emi1*/*p53* morphants, supporting the idea that *cdt1* does not rescue the morphological defects in *emi1* morphants, but rather *emi1* knockdown causes a partial rescue of the pervasive cell death seen in *cdt1* morphants (Fig. S2 A). Indeed, when we injected *cdt1* morpholino in an *emi1* hi2648 clutch, the *emi1* WT embryos showed significant cell death, while the *emi1* mutants exhibited a less severe cell death phenotype (Fig. S2 B). In all, these data suggests that *cdt1* knockdown does not rescue morphological defects in *emi1* morphants. If anything, *emi1* knockdown alleviates some of the cell death in *cdt1* morphants, probably by decreasing the levels of Cyclin A, an inhibitor of *cdt1*, and therefore allowing the little *cdt1* amount present to be active and license replication origins. This hypothesis is also supported by the cell death assay, showing a decreased prevalence of strong Caspase 3 staining in the *emi1 cdt1* double morphants as compared to *cdt1* morphants (Fig. 3 A).

We examined the effects of combinatorial knockdown of *emi1*, *cdt1* and *p53* on the cell cycle distribution. The same amounts of morpholinos were used as in our cell death assay and developmental studies above. A single representative cell cycle distribution is shown in the figures (Fig. 3 B), although experiments were repeated at least 10 times and each experiment had similar results. To compensate for experimental variation, the quantitation of the averages for cell cycle results is shown in Fig. 3D, left panels. As expected, the partial knockdown of *cdt1* resulted in a slight increase in cells with 2 n DNA content and corresponding decrease in cells with >2 n DNA content (Fig. 3 B, D). *Emi1*-deficient cells showed a decrease in 2 n cells and increased cells with 4 n or >4 n DNA content. Despite the lack of *cdt1*-induced rescue of morphological defects in *emi1* morphants, co-depletion of *cdt1* and *emi1* gave rise to an intermediate cell cycle distribution to cells deficient for *emi1* alone *or cdt1* alone. The *emi1*/*cdt1* morphant cells had a modest decrease in the numbers of 2 n cells and a slight increase in the 4 n/>4 n populations compared to control cells. Focusing on the *emi1*-deficient cells, the loss of *cdt1* inhibited the accumulation of >4 n cells and partially rescued the accumulation of 4 n cells in *emi1* morphants (Fig. 3 B, quantitation in Fig. 3 D left panels). The effect of *cdt1* depletion on *emi1* deficiency-induced cell cycle defects was not significantly changed in the presence of *p53* co-knockdown (Fig. 3 B, D). Therefore, we conclude that *cdt1* knockdown effectively rescues cell cycle defects in *emi1-*deficient embryos, in spite of having compounding effects on the embryonic morphology.

We next wanted to investigate whether rereplication accounts for the increased cell size in *emi1*-deficient embryos. It has been suggested that accumulation of more than normal amounts of DNA in a cell correlates with increased nucleus and cell size [11], [12], [13], [18]. As *cdt1* appears to be a major player in the rereplication phenotype in *emi1*-deficient embryos, we wanted to see if *cdt1* knockdown rescues the increased cell size phenotype of *emi1*-deficient cells. We used forward scatter in a FACS assay to determine the cell size in single cell suspensions generated from pools of embryos (Fig. 3 C, D). Cells depleted of both *emi1* and *cdt1* showed an intermediate size, significantly different from *emi1* only, *cdt1* only morphants or control-injected embryos (Fig. 3 C, blue bars). However, when we also knocked down *p53* levels, *cdt1* completely rescued the increased cell size of *emi1* morphants to control levels (Fig. 3 C, red bars).

Interestingly, when we broke down the analysis of cell size by cell cycle stages, it became apparent that *emi1*-deficient cells are significantly larger than controls at all cell cycle stages in the context of *p53*-depletion and the large cell phenotype is not solely due to an increase number of rereplicating large cells (Fig. 3 D). Moreover, *cdt1* loss caused a complete cell size rescue at all cell cycle stages when *p53* was also depleted (Fig. 3 D). As *p53* knockdown efficiently blocks cell death at the embryonic age at which the FACS analysis was performed, the most likely explanation of this data is that *p53* knockdown promotes survival of the smaller cells in *cdt1* and *cdt1/emi1* morphants at all cell cycle stages. Alternatively, *p53* knockdown may have an unexplored effect on the regulation of cell growth.

In conclusion, *cdt1* knockdown completely rescues rereplication and the increased cell size in *emi1* morphants in the context of *p53* depletion, supporting the idea that the large cell phenotype in *emi1*-deficient cells is due to rereplication. However, *cdt1* knockdown does not rescue the developmental defects of *emi1* morphants, not even in the context of *p53* depletion, suggesting there are additional factors that cause morphological defects in *emi1* morphants.

### Decreased Cyclin A and Cyclin B Both Contribute to *emi1*-deficient Phenotype

The complete rescue of *emi1* morphants by knockdown of *cdh1* suggests that the untimely degradation of key Cdh1 targets may be responsible for the defects induced by *emi1* deficiency. Likely candidates include Cyclins A and B [34]. To test this possibility, we tested whether forced expression of Cyclin A and B can rescue *in vivo* any of the phenotypes present in *emi1* morphants. Cyclin A has an established role in origin licensing and S phase entry, as well as mitotic entry (reviewed in [35]). The initiation of Cyclin B expression occurs during S-phase after the onset of Cyclin A expression, and Cyclin B is essential for mitotic entry and progression (reviewed in [36], [37]). However there is limited evidence that suggests a potential role of Cyclin B in replication and S-phase, especially in the absence of Cyclin A activity [38], [39], [40].

**Figure 4:**
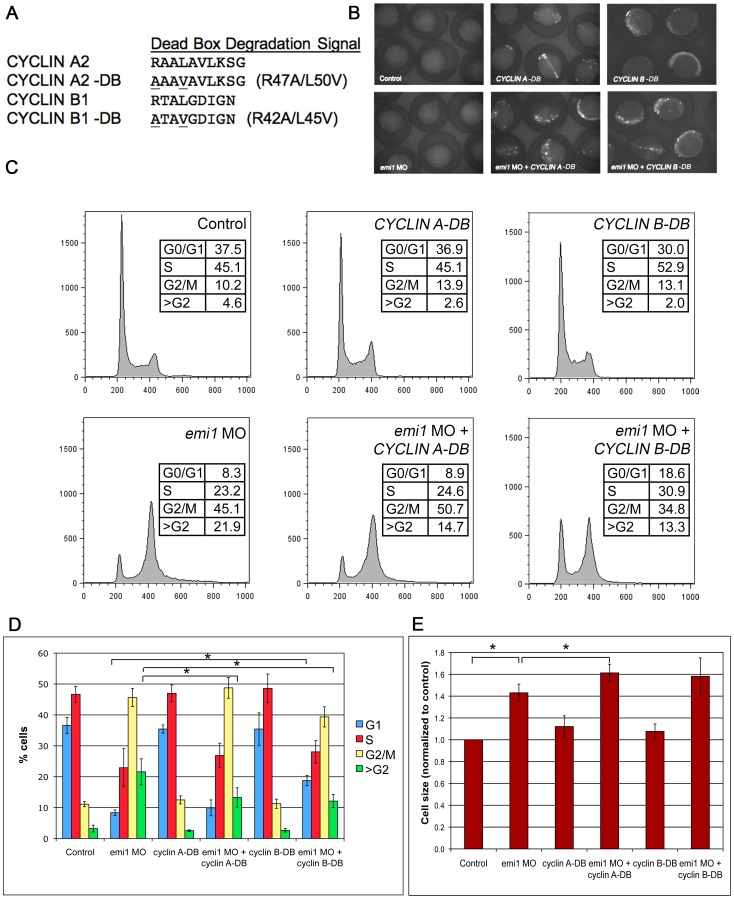
*CYCLIN A2* and *CYCLIN B1* expression partially inhibits rereplication in *emi1*-deficient cells. (A) Amino acid alignment of the dead box domain of wild-type human CYCLIN A2 and CYCLIN B1 and dead box (DB) mutant proteins. (B) Expression of *CYCLIN A*-DB and *CYCLIN B*-DB GFP fusion constructs at 5 somites, as visualized by fluorescence microscopy. Embryos injected with either DNA construct showed significant and pervasive mosaic expression at this stage and the level of expression was not altered by co-injection of *emi1* MO. (C) Representative example of FACS scanning of propidium iodide (PI)-stained total cells from embryos injected with the indicated morpholinos and DNA constructs. Note the partial decrease in >G2 population in embryos injected with *emi1* MO and *CYCLIN A*-DB as compared to *emi1* MO only (quantitation in panel D). Also, embryos injected with *emi1* MO and *CYCLIN B*-DB showed a partially decreased >G2 population and a partially increased G1 population as compared to *emi1* MO only (quantitation in panel D). (D) Quantitation of the cell cycle distribution in embryos injected with the indicated morpholinos and DNA constructs. There was a significant difference between the percentage of cells with >G2 DNA content in embryos injected with *emi1* MO and *CYCLIN A*-DB as compared to *emi1* MO only. Also, there was a significant difference between the percentage of cells with >G2 DNA content and in G1 phase in embryos injected with *emi1* MO and *CYCLIN B*-DB as compared to *emi1* MO only. The results shown are average of 3 independent experiments and error bars indicate standard deviation. (E) Normalized average cell size in embryos injected with the indicated morpholinos and DNA constructs, as indicated by FSC of total cells in the FACS analysis. There was no significant decrease in the size of cells in embryos injected with *emi1* MO and *CYCLIN A*-DB or *CYCLIN B*-DB as compared to *emi1* MO only. If anything, there was a slight but significant increase in cell size of embryos injected with *emi1* MO and *CYCLIN A*-DB as compared to *emi1* MO only.

For our studies, we used human Cyclin A2 and Cyclin B1 because of their established role in cell cycle progression [41]. Cyclins A and B exhibit waves of expression and are targeted for degradation by APC/C ubiquitin ligase [16] at specific steps during cell cycle. We mutated the previously described degradation boxes (DB) [12], [42], [43] to generate CYCLIN A-DB and CYCLIN B-DB proteins that are not recognized by APC/C (Fig. 4 A). The amino acids mutated in the human cyclins are conserved in the zebrafish cyclin homologues. We injected the DNA constructs containing GFP fusions with either *CYCLIN A*-DB or *CYCLIN B*-DB into 1-cell stage zebrafish embryos. The injections resulted in mosaic expression of the GFP fusions at the 5-somite stage (Fig. 4 B).

The forced expression of the non-degradable *CYCLIN A* partially rescued the rereplication defect in *emi1* morphants (Fig. 4 C, D). There is a statistically significant difference between the size of the >G2 populations in *emi1* morphants injected or not with *CYCLIN A-*DB. Our results corroborate previous studies in cell lines demonstrating that a non-degradable form of *CYCLIN A* partially rescued the rereplication defects in cells depleted of *EMI1* [12]. These data are consistent with the established role of *CYCLIN A* in replication initiation and licensing and transition into S-phase.

Surprisingly, forced expression of a non-degradable *CYCLIN B* had an even more profound effect on cell cycle defects in *emi1* morphants: it partially restored the G1 population, but it also rescued the rereplication defect (Fig. 4 C, D). These data provide evidence for a less well-known role of *CYCLIN B* in S-phase and replication [38], [39], [40]. We tested whether *CYCLIN A*-DB and *B*-DB together could have an even more significant rescue on *emi1* morphants. However the injection of both DNA constructs lead to pervasive embryo death (data not shown), preventing our further studies.

Interestingly, the forced expression of *CYCLIN A*-DB or *B*-DB did not rescue the increased cell size phenotype present in *emi1* morphants (Fig. 4 E, S3). This is likely due to the highly mosaic expression of the two constructs in the embryos and the limited number of cells that expressed the Cyclin constructs. Injecting higher levels of *CYCLIN A* and *B* DNA constructs or RNA constructs lead to pervasive embryo death (data not shown), consistent with their essential role in regulating DNA replication and mitosis. In conclusion, we showed for the first time that Cdh1 targets Cyclin A and B can rescue cell cycle defects *in vivo* in *emi1*-deficient embryos, thus providing evidence for the involvement of an Emi1-APC/C-Cdh1-Cyclin A/B axis in suppression of rereplication in vertebrate organisms.

## Discussion

The timely and ordered control of cell cycle is one of the most critical biological processes and understanding the complexity of cell cycle regulation *in vivo* is not an easy task. Here we show for the first time in an *in vivo* vertebrate model that *emi1* disrupts the cell cycle during early embryonic development through an APC/C-Cdh1-dependent mechanism. By studying the rescue effect of inactivating origin licensing on *emi1* depletion, we also establish a novel molecular link between *cdt1* and *emi1* and provide further confirmation that increased cell size correlates with increased cellular DNA content. Lastly, we confirm that *in vivo* Cyclin A is responsible for origin licensing and that Cyclin B appears to have a less-well understood role in replication regulation, on top of the established role in mitotic progression and exit.

### Which APC/C Coactivator is a More Important Target for Emi1 Inhibition *in vivo*?

We took advantage of the prolonged survival of zebrafish embryos deficient in *emi1* as compared to mouse embryos to investigate the molecular pathways that contribute to cell cycle regulation *in vivo*. We showed for the first time in a vertebrate *in vivo* model that knockdown of the APC/C cofactor *cdh1* completely rescued cell cycle defects, including rereplication and G2/M accumulation, and even morphological defects in *emi1*– deficient embryos (Fig. 1, S1). Cdh1 is the APC/C cofactor believed to be important mostly in G1, mitotic exit and G1/S transition in mammalian cells [2], [11], [12], and CDH1 knockdown rescued the cell cycle defects in EMI1-depleted cells [11], [12]. However Cdh1 was suggested to also have an effect in G2 cells in *Drosophila* embryos, where a *cdh1* mutation rescued the mitotic failure of *rca1* (Emi1 homolog) mutants [17].

By contrast, *Xenopus* egg extracts do not express Cdh1, but express Cdc20, the other APC/C activator, which is believed to be more important during mitosis. Cdc20 rescued the mitotic entry block in Emi1-depleted *Xenopus* egg extracts by preventing Cyclin B degradation [1]. However, CDC20 was unstable in mammalian cell lines depleted of *EMI1* [11], probably due to the fact that Cdc20 is also a target of APC/C-Cdh1 late in mitosis [14]. Cdc20 binding to APC/C is controlled by the spindle checkpoint [14] and Cdc20 is essential for mitotic progression and survival in yeast [24], [25] and mice [26]. Consistent with these data, knockdown of *cdc20* was severely lethal in zebrafish embryos (data not shown), which was in stark contrast with *cdh1* knockdown that caused no obvious defects in zebrafish embryos (Fig. 2). It is possible that the embryo survival is due to the fact that the *cdh1* knockdown was not complete (Fig. S1). However, our results are also consistent with *cdh1* not being an essential gene in yeast [44], [45]. In mice, *Cdh1* mutation renders embryonic lethality due to a defect in placentation and, once that defect is rescued by conditional placental *Cdh1* expression, the embryos survive past post natal day 3 [27], suggesting that Cdh1 is not essential for early development in vertebrates.

From these observations, it is unclear whether Cdc20 is an essential target of Emi1 inhibition *in vivo,* mostly due to the difficulty of studying an essential gene like *cdc20 in vivo*. In cell lines, *CDC20* knockdown was required only under some conditions to completely rescue cell cycle defects in *EMI1*-depleted mammalian cells, together with *CDH1* knockdown [12]. Also, the role of Cdc20 as a target of Emi1 inhibition was apparent only in *Xenopus* egg extracts, where Cdh1 was not expressed [46]. In contrast, Cdh1 was the target of Emi1 inhibition in mammalian cells [2], [11], [12], yeast [44], [45] and *Drosophila* [17]. In vertebrates, *cdh1* knockdown completely rescued defects in *emi1*-deficient zebrafish embryos (Fig. 2, S1), suggesting that Cdh1 is the main target of Emi1 inhibition *in vivo*.

### Cdt1, Rereplication and Consequences for Development of *emi1*-deficient Embryos

Rereplication is a major defect observed in *EMI1*-deficient cells and we wanted to investigate the effect of rereplication on embryo development. We showed a complete rescue of the rereplication phenotype in *emi1*-deficient embryos by knockdown of *cdt1*, an important factor that contributes to origin licensing, thus unraveling *cdt1* as a novel effector of *emi1*-induced rereplication. However, rereplication rescue did not cause a rescue of morphological defects in *emi1*-deficient embryos, suggesting that other cell cycle defects such as the block to mitotic entry might have significant consequences to embryo development.

Cdt1 is one of the components of the pre-replicative complex (pre-RC) and it is essential for replication initiation [21], [47], [48]. Once an origin has fired, Cdt1 is phosphorylated by Cyclin A-Cdk and targeted for degradation to prevent origin refiring and hence rereplication [31]. In Emi1-deficient cells, where levels of Cyclin A are low, it is likely that Cdt1 continues to be active and could initiate assembly of other pre-RCs and cause rereplication. We hypothesized that knockdown of *cdt1* might rescue the rereplication defects in *emi1*-deficient embryos. Indeed, when we used a previously published morpholino for *cdt1* [32], we completely rescued the rereplication cells (>4 n cells) in *emi1* morphants back to control levels, even if the *cdt1* knockdown was partial in order to allow embryo survival (Fig. S2, [32]). We even saw a partial but significant rescue of the G2/M population (Fig. 3 B, D). However, we were unable to completely rescue the G2/M population under a variety of *cdt1* morpholino concentrations, implying that there are other factors independent of Cdt1/Cyclin A which are responsible for the block to mitosis in *emi1*-deficient embryos. The rereplication rescue is likely due to the dual roles of *cdt1* in (1) allowing cell cycle progression into S-phase, and, as a secondary effect, the subsequent progression into G2, and (2) the equally important function of *cdt1* in initiating rereplication. Therefore, *cdt1* knockdown prevents DNA replication and causes cells to arrest in G1, so the majority of cells do not even get a chance to transition to G2 and be blocked there upon *emi1* depletion. This scenario is supported by the increased percentage of cells in G1 in *cdt1* morphants and *cdt1*/*emi1* double morphants (Fig. 3 D). It is possible that just a low percentage of cells escape the G1 arrest due to the incomplete knockdown of *cdt1* (Fig. S2), and they get blocked in G2 due to lack of active Emi1. In total, the rereplication rescue exerted by *cdt1* knockdown is likely the combination of a block in the G1 phase of the cell cycle and the inhibition of the initiation of replication/rereplication.

In any case, the complete rescue of rereplication and partial rescue of the G2/M population did not correlate with a rescue of morphological defects in *emi1*-deficient embryos (Fig. S2). It is not clear why loss of *emi1* in the *cdt1*-morphants causes more severe developmental defects than depletion of *cdt1* alone, however this supports the idea that there may be some replication-independent *emi1* roles, such as the regulation of the G2/M transition, that are important in this *in vivo* model or that there are minor disturbances in the replicative machinery that are not detected by the cell cycle assays. Alternatively, it is possible that the partial G1 arrest caused by *cdt1* morpholino is responsible for the disrupted development in the double *cdt1 emi1* morphants.

Because of the significant cell death in *cdt1* morphants and the presence of some cell death in *emi1* morphants (Fig. S2), we tested to see whether p53 activity contributes to the changes in the cell cycle distribution. Co-knockdown of *p53* caused a significant decrease in the amount of cell death, at least at early stages (5 somites, Fig. 3A), supporting a role for p53-dependent cell death upon *cdt1* knockdown. Prolonged inhibition of replication initiation may elicit additional cell death mechanisms that are p53-independent, as indicated by the only partial rescue of cell death at 24hpf (Fig. S2). However, *p53* knockdown did not significantly change the cell cycle distribution for any of the combinations of the *cdt1* or *emi1* morpholinos (Fig. 3 D, left panels), suggesting that the cell cycle distribution, which includes the rereplicating population, in these morphants is p53-independent. It is, however, possible that micro-rereplication sites that do not lead to a detectable increase in DNA content above 4 n could activate p53 and potentially be rescued by p53 knockdown, but this event would not be detected by our FACS analysis. Despite the ability of *p53* depletion to partially rescue *cdt1/emi1* morphant cell size and survival at 5 somites, the co-depleted embryos display cell death and severe developmental defects at 24 hpf. The dysregulated networks and molecular effectors that underlie the developmental defects remain to be determined, including the contribution of p53-independent cell death pathways.

Our data clearly shows a role for Cdt1 supporting rereplication in *emi1*-deficient embryos. This functional interaction between Emi1 and Cdt1 previously escaped notice, perhaps because HeLa cells depleted of *EMI1* showed undetectable levels of CDT1 protein [11]. In this cell line, we postulate that either (1) the levels of CDT1 are sufficient to support rereplication but are not detectable by western blotting or (2) the cells have an alternate mechanism that can support the initiation of replication. Previous studies have shown that Emi1 function is conserved in vertebrates as forced expression of human *EMI1* rescues phenotypes induced by *emi1*-deficiency in zebrafish embryos [13]. Moreover, here we showed *in vivo* that *cdt1* knockdown clearly has an effect on phenotypes induced by *emi1*-deficiency, suggesting that cell-line specific contexts may have prevented previous evaluation of CDT1 in *emi1* deficiency-induced rereplication.

### Is Rereplication the Cause of Increased Cell Size in *emi1*-deficient Embryos?

The size of a cell appears to correlate to some extent with the amount of DNA present in the nucleus, however the causal relationship and regulatory mechanisms that couple these parameters have still not been clearly established [20]. Emi1-depleted cells have an increased DNA content due to rereplication [11], [12], [13], and also appear to have an increased nucleus size as evidenced by DAPI staining [11], [12], [13], [18], and increased cell size as assessed by forward scatter in FACS analysis [13].

We investigated whether the replication phenotype in *emi1*-deficient zebrafish embryos correlates with the increased cell size. *Cdh1* knockdown rescued all cell cycle defects, including rereplication and the increased cell size in *emi1* morphants (Fig. 2). When we focused on origin licensing, we showed that *cdt1* knockdown completely rescues the rereplication defects in *emi1*-deficient zebrafish embryos and we hypothesized that it would also rescue the increased cell size. Across 10 different experiments, *cdt1* knockdown only partially rescued the cell size increase in *emi1* morphants (Fig. 3 C). On the other hand, when p53 was also inactivated, the cell size rescue by *cdt1* morpholino was complete (Fig. 3 C). Our data imply that the cell death present with normal p53 activity may preferentially eliminate smaller cells in the *cdt1* morphants and the *emi1 cdt1* double morphants. Alternatively, it is possible that the apoptotic cells detected by staining of activated Caspase 3 are retained in the FACS assay and have increased size, therefore shifting the average cell size of the *cdt1* morphants and the *emi1 cdt1* double morphants to higher values as compared to the counterparts in the *p53*-deficient background.

In either case, when we analyzed the average cell size in each cell cycle stage, we observed that *emi1*-deficient cells are significantly larger than controls at all cell cycle stages in the context of *p53*-depletion, supporting the idea that large cell phenotype is not solely due to an increased number of rereplicating large cells (Fig. 3 D). These data imply that, while increased DNA content may be the cause of increased cell size in the >G2 population, other mechanisms contribute to the increased cell size in G1, S and G2 cells in *emi1* morphants as compared to control embryos. Therefore, rereplication may not be the sole cause of increased cell size in *emi1*-deficient cells and cell growth regulation may also be important in these cells.

In conclusion, *cdt1* knockdown completely rescues the rereplication phenotype in *emi1* morphants and even the increased cell size of *emi1* morphants in a *p53*-deficient background, but does not rescue morphological defects in *emi1*-deficient embryos, implying that mechanisms independent of rereplication and increased cell size contribute to developmental defects in *emi1*-deficient embryos. Alternatively, it is possible that *emi1* knockdown causes micro-rereplication that does not increase the DNA content above 4 n and would not be detectable by the FACS analysis used in this study. If *cdt1* knockdown does not eliminate rereplication completely and leaves regions of micro-rereplication, this may cause genomic instability [31] and potentially elicit developmental defects.

### Cyclin A and Cyclin B –targets of Inappropriate APC/C Activation in *emi1-*deficient Embryos

APC/C ubiquitin ligase targets Cyclin A and Cyclin B for degradation [43]. In an Emi1-deficient background, it would be predicted that the levels of Cyclin A and B would be low. Therefore we tested whether we could rescue defects in *emi1*-deficient embryos if we augmented Cyclin A or B levels. By injecting non-degradable forms of the Cyclins, we showed for the first time in a vertebrate *in vivo* model that forced expression of Cyclin A partially rescued the rereplication phenotype (Fig. 4 C, D), but not the increased cell size (Fig. 4 E) or the morphological defects (data not shown) in *emi1*-deficient embryos. Our results are consistent with previous studies showing a partial rescue of rereplication of EMI1-depleted cells by expression of non-degradable CYCLIN A [12]. The only *in vivo* data supporting the idea that Cyclin A is a target affected by Emi1 inactivation comes from an experiment in *Drosophila* showing that overexpression of Cyclin A, but interestingly not Cyclin B, rescued the mitotic block imposed by *rca1* mutation in fly embryos [17]. Cyclin A is required for origin licensing at onset of S-phase [30], so our *in vivo* results corroborate the idea that Cyclin A is important to prevent rereplication.

Cyclin B is believed to be important for mitotic progression and exit and we expected that forced expression of a non-degradable form of CYCLIN B in *emi1*-deficient zebrafish embryos would rescue the block to mitosis, but not necessarily the rereplication. Forced expression of CYCLIN B partially restored the G1 population, most likely due to a partial rescue of the mitotic block by pushing cells through and out of mitosis (Fig. 4 C, D). Our *in vivo* data are consistent with studies showing that a non-degradable form of Cyclin B allowed condensation of demembranated DNA in *Xenopus* cycling extracts depleted of Emi1, a prerequisite for mitotic entry [1]. To our surprise though, forced expression of CYCLIN B also partially rescued the rereplication defect in *emi1*-deficient embryos (Fig. 4 C, D), suggesting a role for Cyclin B in control of replication. These results corroborate the idea that Cyclin B may direct replication initiation in the absence of Cyclin A (which would be the case in Emi1-deficient cells), an idea supported by limited evidence so far [38], [39], [40].

The partial rescue of cell cycle defects by forced expression of non-degradable forms of CYCLIN A and B did not correlate with a rescue of cell size (Fig. 4 E, S3) or morphological defects in *emi1*-deficient zebrafish embryos (data not shown). However, the failure of Cyclin A and B to rescue cell size or morphology may reflect the limited number of cells that express these constructs in the embryo.

In conclusion, we show here for the first time that enforced expression of Cdh1 targets Cyclin A and B can rescue cell cycle defects *in vivo* in *emi1*-deficient embryos. Our results support the importance of an Emi1-APC/C-Cdh1-Cyclin A/B axis in suppression of rereplication in vertebrate organisms and open the door for further studies aimed at uncovering other molecular effectors in this axis.

## Materials and Methods

### Ethics Statement and Zebrafish Maintenance

All procedures using experimental animals were approved by the Institutional Animal Care and Use Committee at Fox Chase Cancer Center. Zebrafish adults were bred and embryos were staged using standard practices [49].

### Cloning and Subcloning

Human *CYCLIN A2* (CCNA2)-GFP and *CYCLIN B1* (CCNB1)-GFP constructs have been graciously provided by Dr. Timothy Yen (Fox Chase Cancer Center). Mutations in the cyclin death box [43] for CCNA2 R47A, L50V – (*CYCLIN A*-DB) and CCNB1 R41A, L44V (*CYCLIN B*-DB) were created by using the Agilent Stratagene site-directed mutagenesis kit. *CYCLIN A*-DB and *CYCLIN B*-DB GFP fusions were cloned into pSGH2 heat-shock vector [50]. The pSGH2 heat-shock vector has a dual promoter; heat shock activates expression of the gene of interest and also expression of a reporter GFP construct. We did not heat shock the zebrafish embryos and still obtained significant mosaic expression of the *CYCLIN A*-DB and *CYCLIN B*-DB GFP fusions just front their basal leaky expression.

### Microinjections

Morpholinos (MOs) were obtained from Gene Tools and resuspended in water at a concentration of 1 or 3 mM. Indicated amounts were injected into 1–4 cell stage zebrafish embryos. Morpholinos used are: *emi1* MO -5′ ATTGTCGTTTCACCTCATCATCTG [13]; C-*emi1* MO (5 mismatches indicated in lower case) –5′ATTcTCcTTTCAgCTCATgATgTGA; *cdh1* MO –5′ ATTCCAGATGACAGACTAACCATAG; C-*cdh1* MO (5 mismatches indicated in lower case) – ATTCgAcATcACAcACTAAgCATAG; *cdh1* second MO –5′ GGTACTTTTCTAGGCTCACCTTTTC; *cdt1* MO –5′ TGAGCAGCTATCCTCACCGTTCCTG [32]; C-*cdt1* MO (5 mismatches indicated in lower case) TGAcCAcCTATgCTCAgCcTTCCTG; *p53* MO GCGCCATTGCTTTGCAAGAATTG [51]; C-*p53* MO (5 mismatches indicated in lower case)- 5′ GCaCCATcGCTTgGCAAGcATTG. All morpholinos are splice-blockers and, if the mis-spliced transcripts are stable, they are predicted to cause translation of truncated inactive proteins, except the *p53* morpholino, which blocks ATG-dependent translation.

DNA injections with *CYCLIN A*-DB and *CYCLIN B*-DB GFP fusion constructs were performed in early 1-cell embryos, in the presence of the I-*Sce* I meganuclease to promote DNA integration (as the constructs were cloned into the pSGH2 vector that contains I-*Sce* I restriction sites). The injection cocktails contained the DNA construct (300 ng), I-*Sce* I enzyme reaction buffer (NEB), 4 mM Mg^2+^, 1.2 µL *emi1* morpholino (3 mM stock), 1 µL of I-*Sce* I enzyme (NEB) and phenol red in 5 µL total volume (protocol adapted from [52]). We injected 20 pg of DNA and 2 ng of *emi1* morpholino per embryo.

### FACS Analysis

Cell cycle was analyzed by quantitating incorporation of propidium iodide in disintegrated embryos using flow cytometry, using a modified protocol from [13], [53]. Essentially, 30–100 embryos were dechorinated with pronase, placed in eppendorf tubes and chilled on ice. Embryos were deyolked by pipetting through the tip of a glass pipet and let settle for 1–2 min. The cloudy supernatant was removed and embryos were washed with 1 mL cold FACS buffer for 1 min (10 mM HEPES and 5% FBS in deficient RPMI – without biotin, riboflavin and phenol red). All but 200 µL FACS buffer was removed and embryos were dissociated with pestles, 800 µL FACS buffer was added and samples were filtered through double 85 µm mesh (CMN-0085D from Small Parts) into 5 mL FACS tubes. Samples were washed with additional 2 mL FACS buffer and spun at 1250 rpm, 4°C for 10 min. Supernatant was decanted and cell pellets were resuspended in 100 µL FACS buffer. Cells were fixed by dropwise addition of 2 mL ice cold ethanol 95% while vortexing and incubated for 30 min on ice, after which they were spun at 1250 rpm, 4°C for 10 min. Supernatant was decanted and tubes were dried upside down, wiping the sides with kimwipes. Cells were resuspended in 300–500 µL cocktail of propidium iodide (0.1 mg/mL) and RNase (0.1 mg/mL) in sodium citrate 0.1% and incubated at room temperature for 30 min. Samples were analyzed using a Becton Dickinson FACScan Analyzer and processed using Flowjo software. Mean forward scatter (FSC) was used for cell size analysis. To decrease variation due to sample preparation, control and experimental populations were processed at the same time.

### Caspase 3 Cleavage Assay for Cell Death

We used immunofluorescence detection of cleaved Caspase 3 to assay cell death. Embryos were fixed, transferred in methanol overnight at −20°C, washed 3 times with PBST and preincubated with blocking buffer (20% heat-inactivated FBS, 2% block reagent, 1% DMSO) for one hour at room temperature. Purified polyclonal rabbit antibody anti-active form of Caspase 3 (BD Biosciences, catalog number 559565) was added to a 1∶1000 dilution and embryos were incubated overnight at 4°C. Embryos were washed 4 times with PBST for 15 minutes each and preincubated with blocking buffer (see above) for 1 hour at room temperature. Alexa-488– conjugated anti-rabbit IgM secondary antibody was added to 1∶200 dilution and embryos were incubated for 2 hours at room temperature, then washed 3 times with PBST and mounted for fluorescence microscopy in Vectashield mounting medium. All incubation and washing steps were performed on a nutator.

### Microscopy

Brightfield images were taken using a Nikon SMZ1500 microscope and an Insight color camera and were processed in Adobe Photoshop. Live embryos were immersed in 2% methylcellulose for imaging. Fluorescence imaging was done on a Nikon 80i fluorescent microscope using NIS-Elements software and pictures were taken using a Nikon Digital Sight camera.

## Supporting Information

Figure S1 **Effects of** ***emi1*** **and** ***cdh1*** **morpholinos on RNA splicing and phosphorylation of Histone H2AX (pH2AX).** (A) RT-PCR analysis of RNA from pools of 20 zebrafish embryos following injection with mismatch control (C), *emi1* or *cdh1* morpholinos as indicated. The gene for which RT-PCR was performed is indicated to the left of the panels. The aberrant RT-PCR products indicated were subcloned and verified by sequencing. Both morpholinos were designed to target the exon 2– intron 2 splice-junction and caused deletion of exon 2 (indicated by “del”) and/or partial (“part”) or total insertion of intron 2. RT-PCR of *beta (β)-actin* is a control for RNA quality and quantity. (B) Phospho-Histone H2AX (pH2AX) Western analysis of lysates from pools of embryos injected with the indicated morpholinos. Note the increased amount of pH2AX in *emi1* morphants, which is rescued back to normal levels by *cdh1* knock down.
(TIF)

Figure S2 **Effects of** ***cdt1*** **morpholino on morphology and mRNA splicing.** (A) Brightfield microscopy images demonstrate the morphology of 24-hpf zebrafish embryos after injection of the indicated morpholinos. Note that the knockdown of *p53* alleviates small head and shorter body axis phenotype in *cdt1* and/or *emi1* morphants. (B) Brightfield microscopy images demonstrate the 24-hpf morphological phenotypes due to injecting *cdt1* MO into embryos wildtype or mutant for *emi1*. The quantitation on the right illustrates lack of morphological rescue of emi1 defects by *cdt1* morpholino. (C) *Cdt1* morpholino injection results in aberrant splicing of *cdt1* transcripts (mainly partial insertion of intron 2). RT-PCR analysis was performed with RNA from pools of 20 embryos injected with the indicated morpholinos. Splicing of *cdt1* was analyzed using primers in exon sequences that surround the target exon 3 (top panel). Inclusion of intron 3 in transcripts was assayed using primers targeting exon 3 (forward) and intron 3 (reverse) sequences. Note the (exon 3– intron 3) background product in control MO-injected embryos, which could results from unspliced transcript or genomic DNA contamination. Knockdown of *cdt1* did not affect the *emi1* splicing defects caused by *emi1* morpholino (third panel form top). Co-injection of *p53* morpholino did not alter the splicing patterns of either *cdt1* or *emi1* transcripts. RT-PCR of *beta (β)-actin* was used as a control for RNA quality and quantity (bottom panel).
(TIF)

Figure S3 **Cell size distribution according to cell cycle stages.** Cell size, as indicated by FSC of indicated cell cycle phase populations, was averaged for 3 independent experiments. There was no rescue of increased cell size in *emi1* morphants by co-injection of either *CYCLIN A*-DB or *CYCLIN B*-DB in any of the cell cycle phases.
(TIF)

Methods S1 **Western blots.** Samples were separated on 4–12% polyacrylamide gels, transferred to nitrocellulose and immunoblotted using a rabbit polyclonal antibody anti-zebrafish pH2AX (generous gift of Dr. James Amatruda, University of Texas Southwestern) and anti-actin (AC-40, Sigma; 1∶2000 dilution). Detection was performed using horseradish-peroxidase-conjugated secondary antibodies (Cell signaling; 1∶1000 dilution) and ECL using Immobilon Western Chemiluminescent HRP Substrate (Millipore).
(DOC)
