# Supplementary figures and images for: Rereplication in emi1-Deficient Zebrafish Embryos Occurs through a Cdh1-Mediated Pathway
Source: PLoS One. 2012 Oct 17;7(10):e47658. doi: 10.1371/journal.pone.0047658 (PMC3474755; doi:10.1371/journal.pone.0047658)

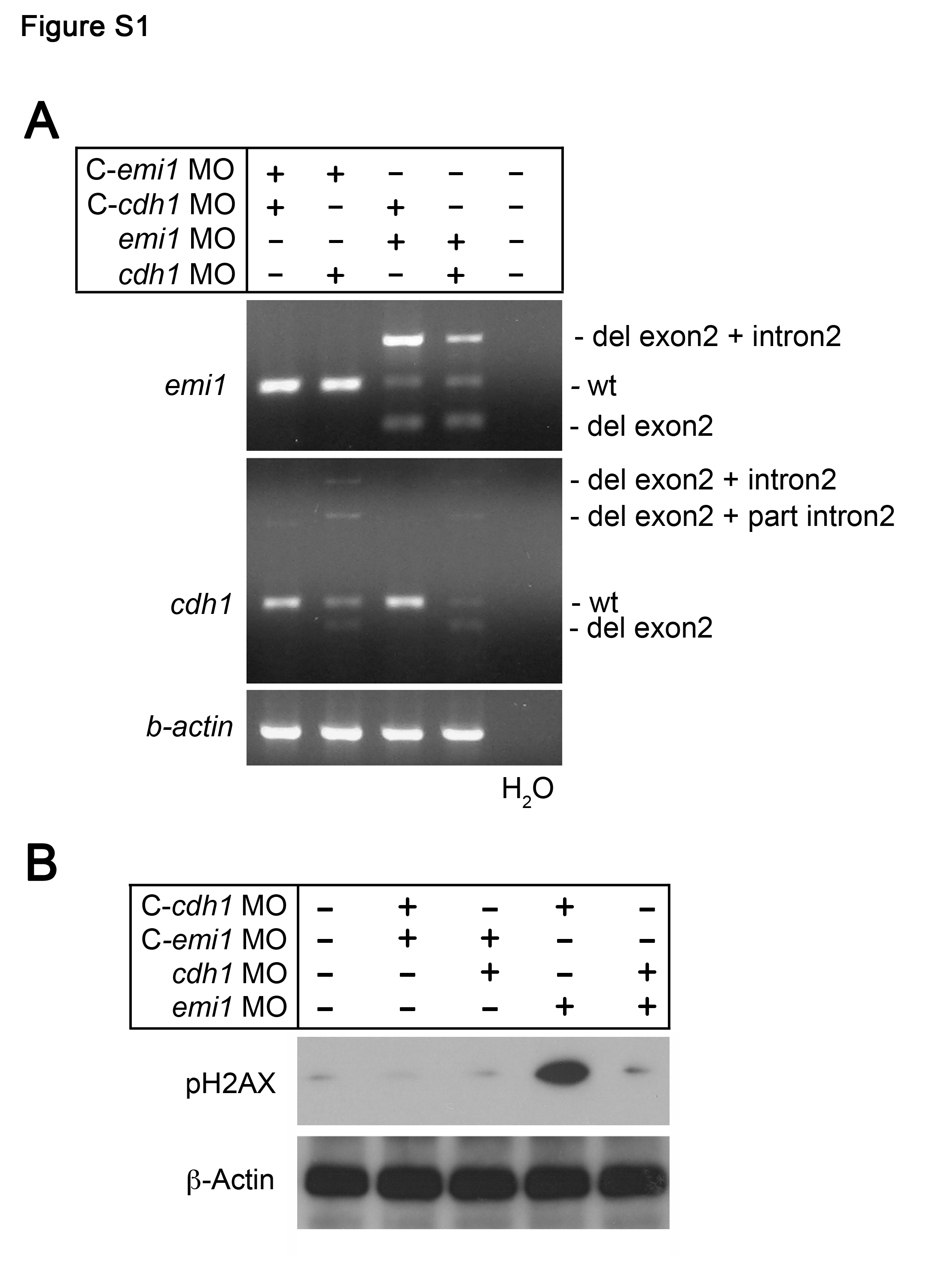

Supplement: Figure S1 — Effects of emi1 and cdh1 morpholinos on RNA splicing and phosphorylation of Histone H2AX (pH2AX). (A) RT-PCR analysis of RNA from pools of 20 zebrafish embryos following injection with mismatch control (C), emi1 or cdh1 morpholinos as indicated. The gene for which RT-PCR was performed is indicated to the left of the panels. The aberrant RT-PCR products indicated were subcloned and verified by sequencing. Both morpholinos were designed to target the exon 2– intron 2 splice-junction and caused deletion of exon 2 (indicated by “del”) and/or partial (“part”) or total insertion of intron 2. RT-PCR of beta (β)-actin is a control for RNA quality and quantity. (B) Phospho-Histone H2AX (pH2AX) Western analysis of lysates from pools of embryos injected with the indicated morpholinos. Note the increased amount of pH2AX in emi1 morphants, which is rescued back to normal levels by cdh1 knock down. (TIF) [file pone.0047658.s001.tif]

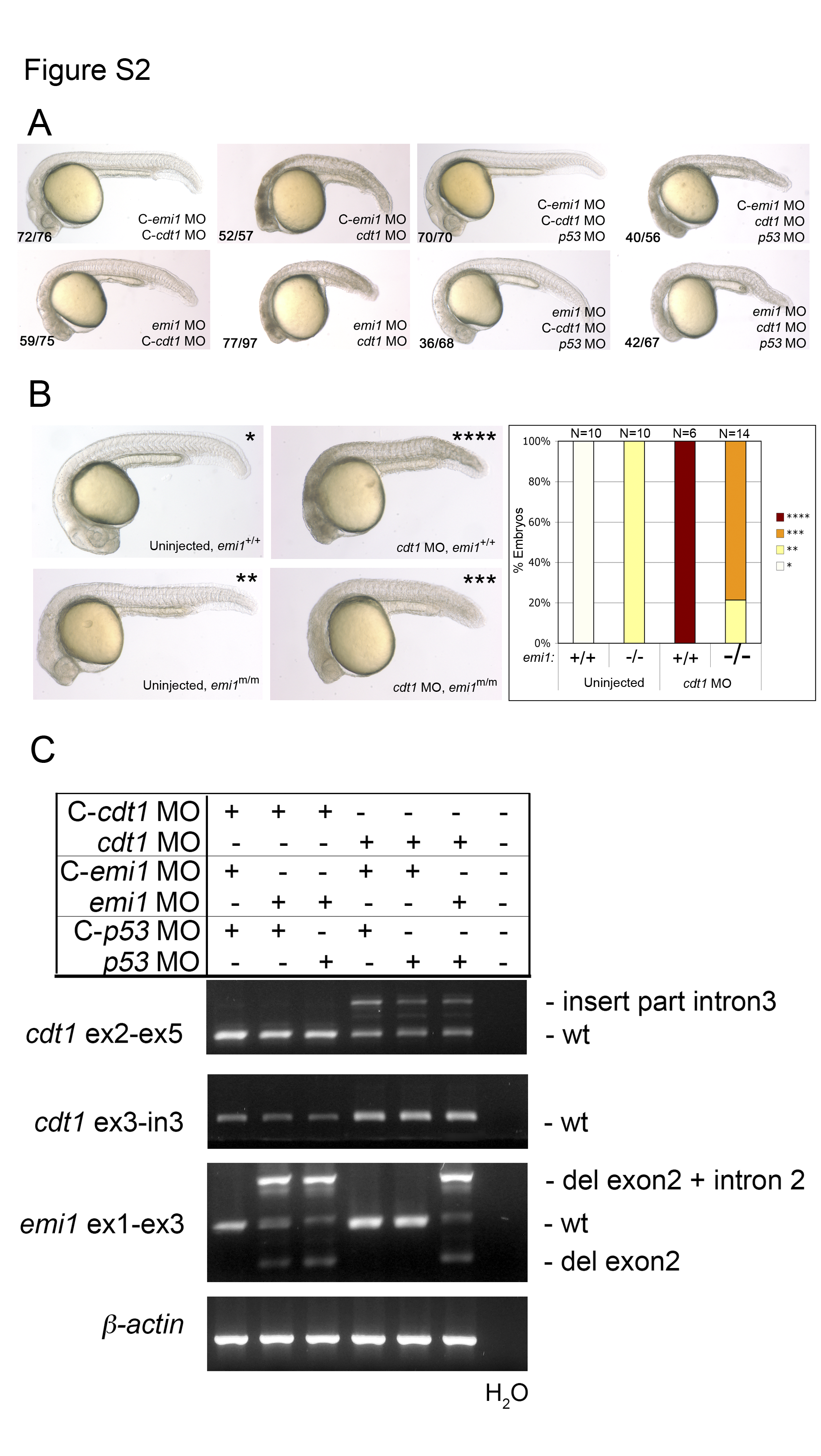

Supplement: Figure S2 — Effects of cdt1 morpholino on morphology and mRNA splicing. (A) Brightfield microscopy images demonstrate the morphology of 24-hpf zebrafish embryos after injection of the indicated morpholinos. Note that the knockdown of p53 alleviates small head and shorter body axis phenotype in cdt1 and/or emi1 morphants. (B) Brightfield microscopy images demonstrate the 24-hpf morphological phenotypes due to injecting cdt1 MO into embryos wildtype or mutant for emi1. The quantitation on the right illustrates lack of morphological rescue of emi1 defects by cdt1 morpholino. (C) Cdt1 morpholino injection results in aberrant splicing of cdt1 transcripts (mainly partial insertion of intron 2). RT-PCR analysis was performed with RNA from pools of 20 embryos injected with the indicated morpholinos. Splicing of cdt1 was analyzed using primers in exon sequences that surround the target exon 3 (top panel). Inclusion of intron 3 in transcripts was assayed using primers targeting exon 3 (forward) and intron 3 (reverse) sequences. Note the (exon 3– intron 3) background product in control MO-injected embryos, which could results from unspliced transcript or genomic DNA contamination. Knockdown of cdt1 did not affect the emi1 splicing defects caused by emi1 morpholino (third panel form top). Co-injection of p53 morpholino did not alter the splicing patterns of either cdt1 or emi1 transcripts. RT-PCR of beta (β)-actin was used as a control for RNA quality and quantity (bottom panel). (TIF) [file pone.0047658.s002.tif]

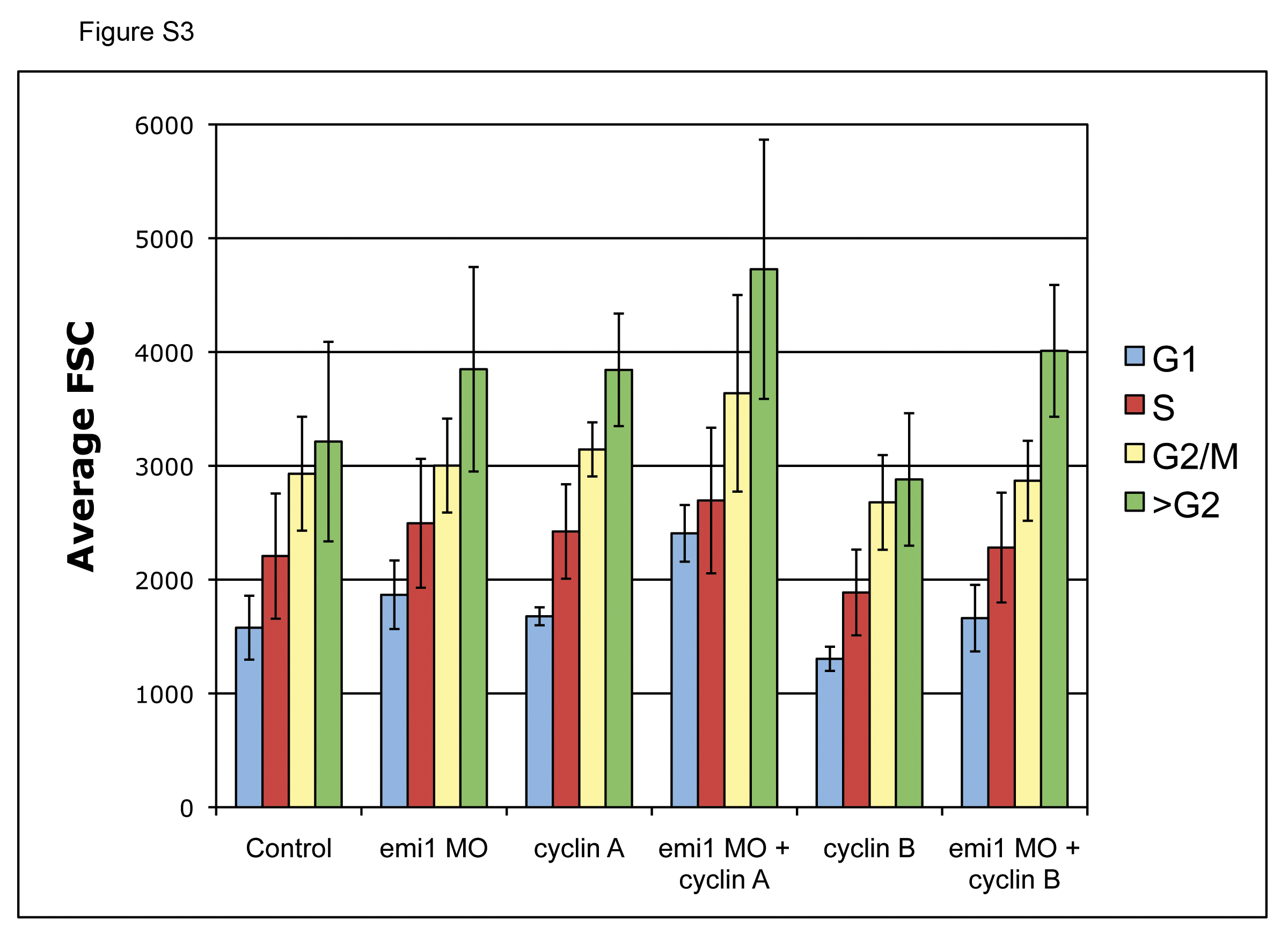

Supplement: Figure S3 — Cell size distribution according to cell cycle stages. Cell size, as indicated by FSC of indicated cell cycle phase populations, was averaged for 3 independent experiments. There was no rescue of increased cell size in emi1 morphants by co-injection of either CYCLIN A-DB or CYCLIN B-DB in any of the cell cycle phases. (TIF) [file pone.0047658.s003.tif]
