# Supplementary material for: Rereplication in emi1-Deficient Zebrafish Embryos Occurs through a Cdh1-Mediated Pathway
Source: PLoS One. 2012 Oct 17;7(10):e47658. doi: 10.1371/journal.pone.0047658 (PMC3474755; doi:10.1371/journal.pone.0047658)
Supplement: Methods S1 — Western blots. Samples were separated on 4–12% polyacrylamide gels, transferred to nitrocellulose and immunoblotted using a rabbit polyclonal antibody anti-zebrafish pH2AX (generous gift of Dr. James Amatruda, University of Texas Southwestern) and anti-actin (AC-40, Sigma; 1∶2000 dilution). Detection was performed using horseradish-peroxidase-conjugated secondary antibodies (Cell signaling; 1∶1000 dilution) and ECL using Immobilon Western Chemiluminescent HRP Substrate (Millipore). (DOC) [file pone.0047658.s004.doc]

**SUPPORTING INFORMATION**

**Figure S1. Effects of *emi1* and *cdh1* morpholinos on RNA splicing and phosphorylation of Histone H2AX (pH2AX).** (A) RT-PCR analysis of RNA from pools of 20 zebrafish embryos following injection with mismatch control (C), *emi1* or *cdh1* morpholinos as indicated. The gene for which RT-PCR was performed is indicated to the left of the panels. The aberrant RT-PCR products indicated were subcloned and verified by sequencing. Both morpholinos were designed to target the exon 2 – intron 2 splice-junction and caused deletion of exon 2 (indicated by “del”) and / or partial (“part”) or total insertion of intron 2. RT-PCR of *beta (β)-actin* is a control for RNA quality and quantity. (B) Phospho-Histone H2AX (pH2AX) Western analysis of lysates from pools of embryos injected with the indicated morpholinos. Note the increased amount of pH2AX in *emi1* morphants, which is rescued back to normal levels by *cdh1* knock down.

**Figure S2. Effects of *cdt1* morpholino on morphology and mRNA splicing.** (A) Brightfield microscopy images demonstrate the morphology of 24-hpf zebrafish embryos after injection of the indicated morpholinos. Note that the knockdown of *p53* alleviates small head and shorter body axis phenotype in *cdt1* and/or *emi1* morphants. (B) Brightfield microscopy images demonstrate the 24-hpf morphological phenotypes due to injecting *cdt1* MO into embryos wildtype or mutant for *emi1*. The quantitation on the right illustrates lack of morphological rescue of emi1 defects by *cdt1* morpholino. (C) *Cdt1* morpholino injection results in aberrant splicing of *cdt1* transcripts (mainly partial insertion of intron 2). RT-PCR analysis was performed with RNA from pools of 20 embryos injected with the indicated morpholinos. Splicing of *cdt1* was analyzed using primers in exon sequences that surround the target exon 3 (top panel). Inclusion of intron 3 in transcripts was assayed using primers targeting exon 3 (forward) and intron 3 (reverse) sequences. Note the (exon 3 – intron 3) background product in control MO-injected embryos, which could results from unspliced transcript or genomic DNA contamination. Knockdown of *cdt1* did not affect the *emi1* splicing defects caused by *emi1* morpholino (third panel form top). Co-injection of *p53* morpholino did not alter the splicing patterns of either *cdt1* or *emi1* transcripts. RT-PCR of *beta (β)-actin* was used as a control for RNA quality and quantity (bottom panel).

**Figure S3. Cell size distribution according to cell cycle stages.** Cell size, as indicated by FSC of indicated cell cycle phase populations, was averaged for 3 independent experiments. There was no rescue of increased cell size in *emi1* morphants by co-injection of either *CYCLIN A*-DB or *CYCLIN B*-DB in any of the cell cycle phases.

**Supplemental Methods**

**Western blots**Samples were separated on 4-12% polyacrylamide gels, transferred to nitrocellulose and immunoblotted using a rabbit polyclonal antibody anti-zebrafish pH2AX (generous gift of Dr. James Amatruda, University of Texas Southwestern) and anti-actin (AC-40, Sigma; 1:2000 dilution). Detection was performed using horseradish-peroxidase-conjugated secondary antibodies (Cell signaling; 1:1000 dilution) and ECL using Immobilon Western Chemiluminescent HRP Substrate (Millipore).
